# Type 1 secretion system and effectors in Rickettsiales

**DOI:** 10.3389/fcimb.2023.1175688

**Published:** 2023-05-15

**Authors:** Duc-Cuong Bui, Tian Luo, Jere W. McBride

**Affiliations:** ^1^ Department of Pathology, University of Texas Medical Branch, Galveston, TX, United States; ^2^ Department of Microbiology and Immunology, University of Texas Medical Branch, Galveston, TX, United States; ^3^ Center for Biodefense and Emerging Infectious Diseases, University of Texas Medical Branch, Galveston, TX, United States; ^4^ Sealy Institute for Vaccine Sciences, University of Texas Medical Branch, Galveston, TX, United States; ^5^ Institute for Human Infections and Immunity, University of Texas Medical Branch, Galveston, TX, United States

**Keywords:** Rickettsiales, *Ehrlichia*, *Orientia*, type 1 secretion system, effector, nucleomodulins, short linear motifs, immunity

## Abstract

Obligate intracellular bacteria in the order Rickettsiales are transmitted by arthropod vectors and cause life-threatening infections in humans and animals. While both type 1 and type 4 secretion systems (T1SS and T4SS) have been identified in this group, the most extensive studies of Rickettsiales T1SS and associated effectors have been performed in *Ehrlichia*. These studies have uncovered important roles for the T1SS effectors in pathobiology and immunity. To evade innate immune responses and promote intracellular survival, *Ehrlichia* and other related obligate pathogens secrete multiple T1SS effectors which interact with a diverse network of host targets associated with essential cellular processes. T1SS effectors have multiple functional activities during infection including acting as nucleomodulins and ligand mimetics that activate evolutionarily conserved cellular signaling pathways. In *Ehrlichia*, an array of newly defined major immunoreactive proteins have been identified that are predicted as T1SS substrates and have conformation-dependent antibody epitopes. These findings highlight the underappreciated and largely uncharacterized roles of T1SS effector proteins in pathobiology and immunity. This review summarizes current knowledge regarding roles of T1SS effectors in Rickettsiales members during infection and explores newly identified immunoreactive proteins as potential T1SS substrates and targets of a protective host immune response.

## Introduction

Members of the order Rickettsiales are Gram-negative obligate intracellular bacteria transmitted by arthropod vectors that cause life-threatening infections in humans and animals (Walker and Ismail, 2008; Renvoisé et al., 2011; Salje, 2021). Rickettsiales are further defined by the Anaplasmataceae and Rickettsiaceae families. The Anaplasmataceae family has two major genera, *Ehrlichia* and *Anaplasma*, that include many pathogens responsible for emerging tick-borne zoonoses (Renvoisé et al., 2011; Salje, 2021). *Orientia* and *Rickettsia* are the major genera of the Rickettsiaceae family and include pathogens responsible for globally distributed life-threatening rickettsioses. The most studied rickettsial pathogens are *R. rickettsii* and *R. prowazekii*, which cause Rocky Mountain spotted fever and louse borne typhus, respectively. *Orientia tsutsugamushi* causes scrub typhus and is transmitted to humans by infected trombiculid mites (Valbuena and Walker, 2013; Walker et al., 2013). Common symptoms associated with rickettsial diseases are non-specific and include headache, fever, myalgia, and localized lymphadenopathy. Diagnostic abnormalities include neutropenia, thrombocytopenia, and moderate increases in transaminases. Currently, there are limited therapeutic options for rickettsial infections, relying primarily on the antibiotic doxycycline, which is most effective with early diagnosis and administration (Paddock and Childs, 2003).

Differences in intracellular life cycles have been described between pathogens in the Anaplasmataceae and Rickettsiaceae families (Salje, 2021). While the Anaplasmataceae family members reside and proliferate within host membrane-derived vacuoles in the infected cell, the Rickettsiaceae family members escape from the endolysosomal pathway shortly after entry and replicate in the host cell cytoplasm. Specifically, Anaplasmataceae members, including *Ehrlichia* and *Anaplasma* spp., share a similar biphasic developmental cycle that involves two morphologically distinct ultrastructural forms, transitioning between an infectious dense-cored cell (DC) and a noninfectious replicating reticulate cell (RC) (Zhang et al., 2007). The DCs attach to a host cell receptor(s) on the surface of target cells through adhesin(s) to facilitate host entry *via* phagocytosis. Once internalized by the host cell, bacteria develop within host cell plasma membrane-derived vacuoles. Within the vacuole, the DC differentiates into the RC, which then multiplies by binary fission every 8 hours to form microcolonies (morulae). The RCs undergo secondary differentiation to form new DCs that egress from the host cell *via* either exocytosis or host cell lysis mechanism.

By contrast, no distinct developmental forms have been described during infection for any members of the Rickettsiaceae family. Further, *Rickettsia* spp. penetrate directly into adjacent cells within a monolayer or lyse heavily infected cells to initiate a new infectious cycle, while *O. tsutsugamushi* utilizes an unusual mechanism that involves budding from the surface of infected cells enveloped in plasma membrane to exit from the host (Salje, 2021). A very recent investigation revealed that *O. tsutsugamushi* bacteria that have budded out of infected host cells were in a distinct developmental stage compared with intracellular bacteria; and these two bacterial stages appeared to differ not only in physical properties, but also in expression of proteins involved in bacterial dormancy, stress response, and outer membrane autotransporter proteins (Atwal et al., 2022). These novel findings suggest that *O. tsutsugamushi* has a biphasic lifecycle like other Anaplasmataceae family members. Genome analyses show that Rickettsiales members have undergone considerable reduction in genome size, ranging from 0.8 to 2.5 Mbp, because of their obligate intracellular lifestyle (Salje, 2021). Intriguingly, Rickettsiales genomes encode various secretion systems homologous to characterized protein secretion pathways found in other bacteria (Gillespie et al., 2014). Notably, the presence of type 1 secretion system (T1SS) components in the genome sequence of many Rickettsiales members suggests that T1SS and related effectors are essential for intracellular infection. Orthologs of *Escherichia coli* T1SS components, including HlyB, HlyD, and TolC, have been identified in *E. chaffeensis*, *A. marginale*, *O. tsutsugamushi*, and *R. typhi* genomes (**Table 1**) (Wakeel et al., 2011; Gillespie et al., 2014; VieBrock et al., 2015; Lin et al., 2021). Although the conservation of these T1SS components is documented in Rickettsiales, there is limited knowledge of how T1SS functions in their pathogenesis. To date, most knowledge on the Rickettsiales T1SS and associated effectors has been revealed through investigations of *E. chaffeensis* and *O. tsutsugamushi* (Wakeel et al., 2011; VieBrock et al., 2015) (**Table 2**). These studies have identified and experimentally demonstrated multiple T1SS effector substrates in both pathogens (Wakeel et al., 2011; VieBrock et al., 2015). These T1SS substrates are bacterial effectors with moonlighting, or multiple functions that directly engage and manipulate a diverse array of host cellular targets to promote infection and evade innate immune responses (Zhu et al., 2009; Zhu et al., 2011; Dunphy et al., 2014; Farris et al., 2016; Beyer et al., 2017; Zhu et al., 2017; Evans et al., 2018; Kibler et al., 2018; Klema et al., 2018; Luo et al., 2018; Rodino et al., 2018; Wang et al., 2020; Adcox et al., 2021; Rogan et al., 2021; Byerly et al., 2022; Patterson et al., 2022).

**Table 1:** Defined type 1 secretion system (T1SS) components in Rickettsiales members.

Family	Species	Protein	Locus ID/Accession	*E. coli* identity	Reference
Anaplasmataceae	*Ehrlichia chaffeensis*	HylB	ECH_0383	27%	(Wakeel et al., 2011)
		HylD	ECH_0970	28%	(Wakeel et al., 2011)
		TolC	ECH_1020	26%	(Wakeel et al., 2011)
	*Anaplasma marginale*	HylB	YP_154094	26%	(Gillespie et al., 2014)
		HylD	YP_154094	27%	(Gillespie et al., 2014)
		TolC	AAV86905	20%	(Gillespie et al., 2014)
Rickettsiaceae	*Orientia tsutsugamushi*	HylB	OTT_1108	41%	(VieBrock et al., 2015)
		HylD	OTT_1107	27%	(VieBrock et al., 2015)
		TolC	OTT_0076	22%	(VieBrock et al., 2015)
	*Rickettsia typhi*	HylB	RT0305	21%	(Kaur et al., 2012)
		HylD	RT0304	24%	(Kaur et al., 2012)
		TolC	RT0216	23%	(Kaur et al., 2012)

**Table 2:** Defined T1SS substrates in Rickettsiales members.

Species	Protein	Locus ID/Accession	Function	Reference
*Ehrlichia chaffeensis*	TRP32	ECH_0170	Nucleomodulin that directly regulates expression of host genes governing differentiation and proliferation	(Wakeel et al., 2011; Farris et al., 2016)
	TRP47	ECH_0166	Nucleomodulin that enters the nucleus *via* a MYND-binding domain and plays a role in host cell reprogramming by regulation of transcription	(Wakeel et al., 2011; Kibler et al., 2018)
	TRP75	ECH_0558	Interacts with host cell targets involved in homeostasis, cytoskeleton organization, and apoptosis regulation to promote infection	(Luo et al., 2018)
	TRP120	ECH_0039	Moonlighting functions, including as molecular mimicry, nucleomodulin, DNA binding, a SUMO/HECT E3 ligase involved in self- and host ubiquitination to influence protein interactions and stability for intracellular survival	(Wakeel et al., 2011; Zhu et al., 2011; Dunphy et al., 2014; Zhu et al., 2017; Klema et al., 2018; Wang et al., 2020; Byerly et al., 2021; Rogan et al., 2021; Byerly et al., 2022; Patterson et al., 2022)
	Ank200	ECH_0684	Nucleomodulin that binds adenine-rich *Alu* elements in host promoter and intron regions	(Zhu et al., 2009; Wakeel et al., 2011)
*Orientia tsutsugamushi*	Ank1	OTT_0753	Modulates NF-κB p65 nuclear transport and inhibits NF-κB transcriptional activation	(VieBrock et al., 2015; Evans et al., 2018)
	Ank2	OTT_0049	ND^a^	(VieBrock et al., 2015)
	Ank3	OTT_1112	ND	(VieBrock et al., 2015)
	Ank4	OTT_0210	Modulates endoplasmic reticulum-associated degradation to benefit bacterial growth	(VieBrock et al., 2015; Rodino et al., 2018)
	Ank5	OTT_0214	ND	(VieBrock et al., 2015)
	Ank6	OTT_1149	Modulates NF-κB p65 nuclear transport and inhibits NF-κB transcriptional activation	(VieBrock et al., 2015; Evans et al., 2018)
	Ank7	OTT_1509	ND	(VieBrock et al., 2015)
	Ank8	OTT_0257	ND	(VieBrock et al., 2015)
	Ank9	OTT_0298	Utilizes a novel GRIP-like Golgi localization domain for Golgi-to-endoplasmic reticulum trafficking and interacts with host COPB2	(VieBrock et al., 2015; Beyer et al., 2017)
	Ank10	OTT_0398	ND	(VieBrock et al., 2015)
	Ank11	OTT_0459	ND	(VieBrock et al., 2015)
	Ank12	OTT_0602	ND	(VieBrock et al., 2015)
	Ank13	OTT_0852	Nucleomodulin that exploits the RaDAR nuclear import pathway to reprogram host cell transcription	(VieBrock et al., 2015; Adcox et al., 2021)
	Ank14	OTT_1019	ND	(VieBrock et al., 2015)
	Ank15	OTT_1232	ND	(VieBrock et al., 2015)
	Ank17	OTT_1478	ND	(VieBrock et al., 2015)
	Ank18	OTT_1518	ND	(VieBrock et al., 2015)
	Ank19	OTT_1519	ND	(VieBrock et al., 2015)
	Ank20	OTT_1575	ND	(VieBrock et al., 2015)

a Not determined.

A growing body of evidence demonstrates that *Ehrlichia* T1SS effectors are moonlighting proteins that exploit and modulate host cell processes, including cytoskeletal organization, vesicular trafficking, cell signaling, transcriptional regulation, post-translational modifications, autophagy, and apoptosis (Byerly et al., 2021). Notably, multiple *Ehrlichia* and *Orientia* T1SS effectors act as bacterial nucleomodulins, a novel class of bacterial effectors that can enter and modulate various nuclear activities (Wakeel et al., 2011; Zhu et al., 2011; Farris et al., 2016; Evans et al., 2018; Kibler et al., 2018; Adcox et al., 2021). Further, recent studies determined that *E. chaffeensis* activates distinct conserved signaling pathways such as Wnt, Notch, and Hedgehog (Hh) *via* ligand mimicry (Byerly et al., 2021; Rogan et al., 2021; Byerly et al., 2022; Patterson et al., 2022). These relatively recent discoveries illuminate important roles of T1SS effectors in the pathobiology of obligate intracellular bacteria.

## Features of the T1SS and effectors

**Figure 1:**
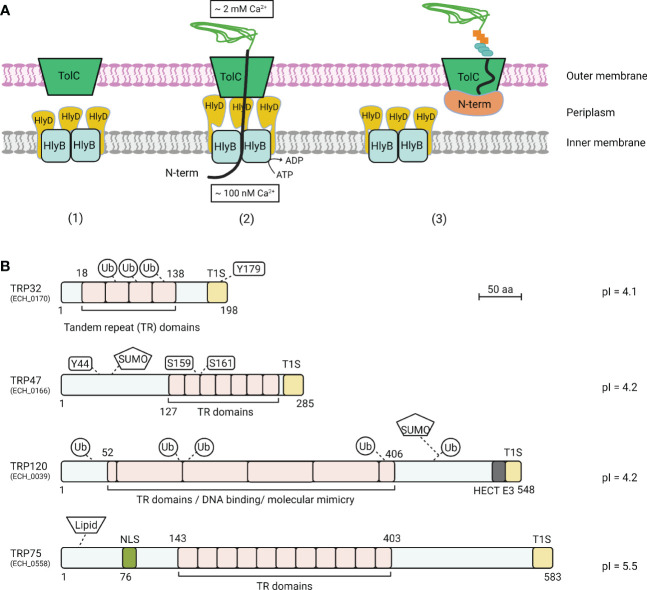
T1SS model and *E. chaffeensis* T1SS TRP effectors. **(A)** *Escherichia coli* T1SS components are typically composed of an ATP-binding cassette protein (HlyB), a membrane fusion protein (HlyD), and a bacterial outer membrane protein (TolC). (1) A T1SS consists of HlyB inner membrane and HlyD periplasm complex and a TolC outer membrane channel. (2) In the classic mechanism of protein translocation by T1SS, protein substrate association with HlyB triggers the interaction of HlyD with TolC channels in the outer membrane, followed by direct protein secretion. (3) In the stalled translocation T1SS model, the N-terminal domain starts folding prior to or during secretion, which plugs the translocon and tethers the entire substrate at the cell surface within the outer membrane component of the T1SS translocon. **(B)** Schematic of *E. chaffeensis* T1SS TRPs and important features. TRPs contain molecularly distinct TR domains that vary in sequence, length, and number. TRPs are highly acidic, contain type 1 secretion (T1S) signal sequences in the C-terminal domain. Multiple post-translational modifications, including phosphorylation, ubiquitination, and SUMOylation have been identified on TRP effectors during ehrlichial infection. TRPs have multiple functions including acting as nucleomodulins that modulate host gene transcription, HECT E3 ubiquitin ligase activity, and ligand mimetic activity.

The T1SS is widespread among Gram-negative bacteria and is especially common in extracellular pathogenic bacteria. It acts as a dedicated nano machine to direct the translocation of various proteins with diverse characteristics and functions from the bacterial cytoplasm to the extracellular milieu (Delepelaire, 2004). In *E. coli*, the T1SS consists of three indispensable structural components: an ATP-binding cassette protein (HlyB) that resides in the bacterial inner membrane, a periplasmic-spanning membrane fusion protein (HlyD), and a bacterial outer membrane protein (TolC) (**Figure 1A**) (Delepelaire, 2004). Secretion of an unfolded T1SS substrate protein occurs when the C-terminal secretion signal is recognized by a homodimer formed by HlyB, which then hydrolyzes ATP to mediate translocation of the substrate protein across the inner membrane into the HlyD trimeric pore. HlyD subsequently spans the entire periplasm and interacts with TolC in the outer membrane, resulting in formation of a transient HlyB-HlyD-TolC complex. This complex consequently serves as a channel that spans the entire distance from the bacterial cytoplasm across the inner membrane, periplasm, and outer membrane to the extracellular space, thereby mediating one-step translocation of bacterial proteins (Delepelaire, 2004; Holland et al., 2005). A less common molecular mechanism underlying T1SS protein translocation involves a two-step process and was recently characterized in *Pseudomonas fluorescens* secretion of adhesin LapA (Spitz et al., 2019). In addition to the C-terminal T1SS signal sequence, LapA also possesses an N-terminal retention module (RM) that anchors the adhesin to the cell surface to stall further translocation (Smith et al., 2018). Specifically, the RM preserves an interaction between LapA and TolC, which results in LapA being anchored to the outer membrane where it functions as an adhesion to facilitate biofilm formation. During unfavorable conditions that affect biofilm formation, the RM is removed by proteolysis, releasing LapA from the bacterial outer membrane. This novel finding highlights the diversity and flexibility of the bacterial T1SS and associated effectors to actively establish a permissive niche for survival.

Various T1SS substrate proteins have been identified as exotoxins, adhesins, S-layer proteins (proteins that form 2-dimensional macromolecular structures on the surface of bacteria), and heme-binding proteins (Green and Mecsas, 2016). The most studied T1SS substrates, the repeats-in-toxins (RTX) family exoproteins, harbor two indispensable regions that determine translocation by the T1SS. First, the noncleavable T1SS signal sequence is located at the final carboxy-terminal 50- to 100- residues and is rich in several amino acids (e.g., leucine, aspartic acid, alanine, valine, threonine, serine, isoleucine, phenylalanine) and poor in others (e.g., lysine, histidine, proline, methionine, tryptophan, cysteine) (Delepelaire, 2004; Wakeel et al., 2011; Thomas et al., 2014). These T1SS signal sequences can initiate secretion of the substrate protein by interacting with the T1SS translocase HlyB-HlyD complex, followed by secretion of the substrate protein through the HlyB-HlyD-TolC complex as described above. Second, the Ca^2+^-binding nonapeptide repeats, so-called GG repeats, are located proximal to the T1SS signal sequences. The GG repeats appear to play a crucial role in enhancing secretion efficiency of T1SS substrate proteins. Once exported to the extracellular milieu, the GG repeats bind to Ca^2+^ ions to form a stable β-roll, creating steric hindrance to prevent the protein from sliding back into the bacterial cell by Brownian motion (Bumba et al., 2016). Therefore, the intracellular/extracellular calcium gradient is important relative to successful translocation of T1SS substrate proteins (Guo et al., 2019). Notably, a recent investigation in *E. coli* determined that the RTX domain of the T1SS substrate HlyA, which harbors the GG repeat and the T1SS signal region at the extreme C-terminus, did not require a defined sequence to regulates HlyA secretion (Spitz et al., 2022). Rather, a putative amphipathic α-helix in the C-terminus region of HlyA was demonstrated to play essential role in the early steps of the secretion process (Spitz et al., 2022). This compelling evidence supports the current model proposed that secondary structures might be encoded in the secretion signal of T1SS effectors. Further, the T1SS substrate proteins have been shown to have acidic isoelectric points (pIs) and contain few or no cysteine residues (Wakeel et al., 2011; VieBrock et al., 2015).

Investigation of T1SS and related substrates in the Rickettsiales, including *Ehrlichia* and *Orientia*, has not been performed due to the lack of genetic tools available (Cheng et al., 2013; Wang et al., 2017; Adcox et al., 2021). Thus, *Ehrlichia* and *Orientia* T1SS and associated effectors have been identified using heterologous *E. coli* T1SS (Wakeel et al., 2011; VieBrock et al., 2015). Over the course of infection, studies in *E. chaffeensis* tandem repeat containing protein (TRP) T1SS effectors using immunoelectron microscopy revealed that these TRPs extracellularly associated with morular fibrillar matrix and the morula membrane, indicating the secretion of these proteins into the *E. chaffeensis*-containing vacuole (Popov et al., 2000; Doyle et al., 2006; Luo et al., 2008). The underlying molecular mechanism of how the T1SS effectors from vacuole bound rickettsial pathogens translocating from the lumen vacuole to the host cytoplasm has not been investigated. However, investigations have defined various mechanisms by which these T1SS effectors traffic to the host nucleus during infection to act as nucleomodulins. For example, study in *E. chaffeensis* T1SS effectors has shown that TRP47 utilizes a MYND (Myeloid Nervy DEAF-1) domain to translocate to the host nuclear (Kibler et al., 2018). While *O. tsutsugamushi* Ank1 and Ank6 localize to the nucleus in an importin β1-dependent manner, *O. tsutsugamushi* Ank13 co-opts eukaryotic RaDAR (RanGDP-ankyrin repeats) nuclear import (Evans et al., 2018; Adcox et al., 2021). In addition, newly identified immunoreactive proteins from *E. chaffeensis* and *E. canis* ORFeomes (complete set of open reading frames) are also predicted to be secreted by the T1SS and are discussed in greater detail below (**Table 3**) (Luo et al., 2020; Luo et al., 2021), highlighting the largely uncharacterized roles of T1SS components and substrates in pathobiology and immunity to rickettsial pathogens.

**Table 3:** Major immunoreactive proteins of *E. chaffeensis* and *E. canis* that are predicted T1SS substrates.

Species	Protein (ECH/ECAJ_tag no.)	Molecular weight (kDa)	Trans-membrane domains^b^	Effector^c^	Annotation
*E. chaffeensis*	ECH_1053	small^a^	+	+	hypothetical protein
	ECH_0846	small		+	hypothetical protein
	ECH_0745	small		+	hypothetical protein
	ECH_0700	small		+	hypothetical protein
	ECH_0607	38		+	hypothetical protein
	ECH_0678	small	+	+	hypothetical protein
	ECH_0207	small	+	+	hypothetical protein
	ECH_0673	28	+	+	hypothetical protein
	ECH_1128	small	+	+	hypothetical protein
	ECH_0706	small		+	hypothetical protein
	ECH_0635	39	+	+	hypothetical protein
	ECH_0988	small	+	+	hypothetical protein
	ECH_0640	32		+	hypothetical protein
*E. canis*	ECAJ_0919	small		+	hypothetical protein
	ECAJ_0717	small	+	+	hypothetical protein
	ECAJ_0920	small		+	hypothetical protein
	ECAJ_0676	small		+	hypothetical protein
	ECAJ_0922	small		+	hypothetical protein
	ECAJ_0151	small	+	+	electron transport protein SCO1/SenC
	ECAJ_0128	39	+	+	extracellular solute-binding protein, family 1
	ECAJ_0104	48	+	+	hypothetical protein
	ECAJ_0179	small		+	formylmethionine deformylase
	ECAJ_0589	41		+	DNA-directed RNA polymerase subunit alpha
	ECAJ_0850	51		+	insulinase-like peptidase M16, C-terminal
	ECAJ_0818	small		+	LexA family transcriptional regulator

a Protein with ≤ 250 amino acids.

b Predicted by TMHMM; +, harbor transmembrane domain.

c Predicted by PREFFECTOR; +, function as an effector.

Genomic studies reveal the presence of T1SS apparatus orthologs in the genome sequences of many obligate intracellular bacteria in the order Rickettsiales (**Table 1**) (Wakeel et al., 2011; Kaur et al., 2012; Gillespie et al., 2014; VieBrock et al., 2015; Lin et al., 2021). *In silico* analysis revealed that the identity to *E. coli* of these T1SS components in Rickettsiales members varies, ranging from 20-40% in *E. chaffeensis*, *A. marginale*, *O. tsutsugamushi* and *R. typhi* (**Table 1**). Functional investigations further demonstrated the feasibility of *E. coli* system as a heterologous host for studying T1SS and effectors in different members of Rickettsiales, including *E. chaffeensis* and *O. tsutsugamushi* (Wakeel et al., 2011; VieBrock et al., 2015), suggesting that *E. coli* system might also be applicable for studying T1SS and effectors in other obligate intracellular bacteria. Despite conservation of these T1SS components, investigation of the T1SS and related effectors among obligate intracellular pathogens in the Rickettsiales has been somewhat overshadowed by investigations of another important effector secretion system that is more often associated with pathogenicity and virulence, the type 4 secretion system (T4SS). As such, in the last decade, the function of T1SS and associated effector proteins has been characterized only in *E. chaffeensis* and *O. tsutsugamushi* (Wakeel et al., 2011; VieBrock et al., 2015). *E. chaffeensis* T1SS effectors, including TRP32, TRP47, TRP120, and Ank200, were first identified a decade ago as T1SS substrates. These *E. chaffeensis* T1SS effectors are related to the RTX exoprotein family, which are common proteins in Gram-negative bacteria containing glycine- and aspartate-rich tandem repeats (Wakeel et al., 2011). The heterologous expression system *E. coli* K-12 strain BW25113 that contains *tolC*, but not *hlyCABD* genes required for secretion of T1SS substrate hemolysin, was utilized for identifying *E. chaffeensis* T1SS substrate effectors, including TRP32, TRP47, TRP120, and ANK200. Specifically, the secretion of full length TRP32, TRP47, TRP120, and the C-terminal 112 amino acids of ankyrin repeat 200 (ANK200C) protein was detected in the extracellular medium of the *E. coli* hemolysin T1SS only in the presence of vector expressing HlyBD. These results demonstrated that TRP32FL, TRP47FL, TRP120FL, and Ank200C4 were secreted by a functional T1SS system. Further, confirming the importance of the secretion signal sequences in the C-terminal domain of the T1SS substrate proteins, the secretion of TRP47 C-terminal, but not TRP47 N-terminal, was detected in the extracellular medium using the similar system. These studies also demonstrated the importance of TolC in the secretion of ehrlichial effectors as a *tolC210::Tn10* (an insertional mutant derivative of *E. coli* K-12 strain CAG12184) mutant strain had reduced secretion of TRP32FL, TRP47FL, TRP120FL, and Ank200C4 (Wakeel et al., 2011). Subsequently, *O. tsutsugamushi* T1SS substrate proteins were also identified (VieBrock et al., 2015). Due to the toxicity in *E. coli* C600 of full-length *O. tsutsugamushi* Ank proteins, another heterologous system was developed for assessing *O. tsutsugamushi* Anks as potential T1SS substrates using *E. coli* BL21 (DE3). To reduce toxicity of full-length proteins, the 60 C-terminal residues which should contain the T1SS signal sequence of *O. tsutsugamushi* Ank proteins was replaced with the C-terminal 60 amino acids of HlyA and subsequently tested for secretion in an HlyBD-dependent manner. C-terminal domains of *O. tsutsugamushi* Ank proteins tested (19 of 20) were secreted in a HlyBD-dependent manner, suggesting *O. tsutsugamushi* Ank proteins are T1SS effector proteins (VieBrock et al., 2015).

Another distinguishing aspect of ehrlichial T1SS effectors is that T1SS effectors elicit strong host antibody responses during infection. Specifically, investigations over three decades have molecularly characterized a subset of immunoreactive *Ehrlichia* T1SS effectors, including ankyrin (Ank) and tandem repeat proteins (TRPs), that have molecularly defined linear antibody epitopes (Doyle et al., 2006; McBride et al., 2007; Luo et al., 2008; Luo et al., 2009; McBride et al., 2011). Strong TRP- and Ank-specific antibody responses are observed in humans and dogs during infection, and antibodies directed at linear epitopes of *E. chaffeensis* TRP proteins are protective against ehrlichial infection (Chen et al., 1997; Li et al., 2001; McBride et al., 2003; Kuriakose et al., 2012). Notably, recent advances have identified novel repertoires of undiscovered major immunoreactive proteins from *E. chaffeensis* and *E. canis* ORFeomes (complete set of open reading frames of the genome), many of which are predicted to be T1SS effectors with confirmation-dependent antibody epitopes (Luo et al., 2020; Luo et al., 2021). These findings reveal the underappreciated and largely uncharacterized roles of the T1SS effector proteins in immunity to *Ehrlichia* and related obligates.

Understanding the molecular roles of the Rickettsiales T1SS effectors in pathobiology and immunity is essential for developing therapeutics against defined mechanistic targets. Further, identifying major immunoreactive proteins of rickettsial pathogens may provide new options for effective subunit vaccines. This work aims to provide an overview of the T1SS and molecularly defined T1SS effectors in obligate intracellular bacteria in the order Rickettsiales. We will highlight the moonlighting functions of the most well defined T1SS effectors in *E. chaffeensis* and *O. tsutsugamushi* and recent findings demonstrating that ehrlichial effectors are major targets of the host immune response. The following subtopics will focus on *E. chaffeensis* and *O. tsutsugamushi* T1SS effectors and recent new knowledge regarding the molecular roles of T1SS effectors in pathobiology and immunity that provide a foundation for further investigation of these and other obligate intracellular bacterial pathogens.

### Introduction to *Ehrlichia* T1SS effectors

The molecular functions connected to T1SS effector substrates in intracellular bacterial pathobiology and the potential relationship with immunity have been most extensively investigated in *E. chaffeensis*. Bioinformatic analysis of *E. chaffeensis* TRP32, TRP47, TRP75, TRP120, and Ank200 revealed key features consistent with those typically described in the RTX family of exoproteins, including glycine- and aspartate-rich tandem repeats, acidic pIs, and noncleavable type 1 secretion signal sequences containing the LDAVTSIF-enriched motif in the C-terminal region (**Figure 1B**) (Wakeel et al., 2011). During infection, *E. chaffeensis* T1SS effectors, including TRP32, TRP47, TRP120, and Ank200, are translocated to the host cell nucleus where they are involved in reprogramming host cell transcription to accommodate intracellular infection (**Table 2**) (Byerly et al., 2021). TRP75 interacts with multiple different eukaryotic proteins that are localized in the cytosol (Luo et al., 2018; Byerly et al., 2021). Ultimately, ehrlichial T1SS effectors fall into one of two categories: host cell signaling ligand mimetics and nucleomodulators. As detailed in a following section, the *Ehrlichia* T1SS effector proteins demonstrate the sophisticated mechanisms used to establish a permissive environment for infection, while evading innate host defenses. Therefore, understanding molecular and protective roles of the newly predicted *Ehrlichia* T1SS effectors is a high priority and may lead to new options for effective therapeutics and subunit vaccines.

### Introduction to *Orientia tsutsugamushi* T1SS effectors

A total of 19 *O. tsutsugamushi* Ank effectors have been identified as T1SS substrates, while only Ank1, Ank6, Ank9, and Ank13 have been molecularly and functionally characterized (**Table 2**). Specifically, *O. tsutsugamushi* employs Ank1 and Ank6 to inhibit accumulation of nuclear factor kappa-light-chain-enhancer of active B cells (NF-κB) and NF-κB-dependent transcription during infection (Evans et al., 2018). Ank9 appears to be a multifunctional *O. tsutsugamushi* T1SS effector (Beyer et al., 2017). During infection, Ank9 utilizes a novel GRIP-like Golgi localization domain for Golgi-to-endoplasmic reticulum trafficking (Beyer et al., 2017). Further, Ank9 has been shown to interact with host COPB2 (coatomer protein complex subunit [COPI] beta 2) which mediates Golgi-to-endoplasmic reticulum transport (Beyer et al., 2017). *O. tsutsugamushi* Ank13 functions as a nucleomodulin during infection (Adcox et al., 2021). Mechanistically, Ank13 exploits the RanGDP-ankyrin repeat (RaDAR) nuclear import pathway to enter the nucleus and modulate host cell transcription (Adcox et al., 2021). These interesting examples illuminate the important role of T1SS effectors in pathobiology of *O. tsutsugamushi* and support functional characterization of other identified T1SS Ank effectors.

## Host cell signaling pathway modulation and ligand mimetics

**Figure 2:**
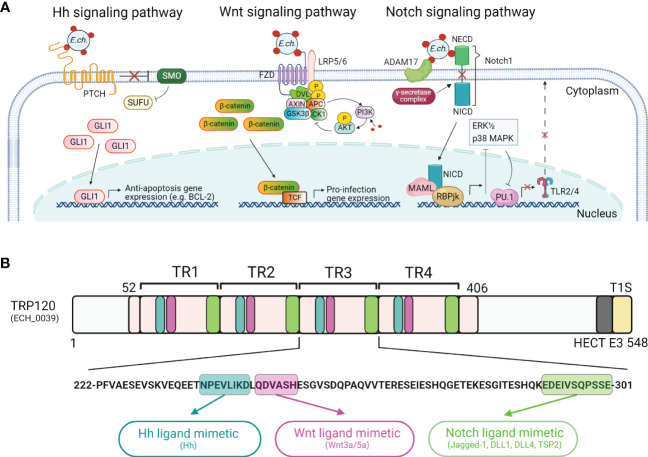
*Ehrlichia* SLiM ligand mimicry activation of conserved signaling pathways. **(A)** Proposed model of *E. chaffeensis* manipulation of Hedgehog (Hh), Wnt, and Notch signaling pathways by T1SS effector, TRP120. For the Hh signaling pathway, dense-core *E. chaffeensis* surface-expressed TRP120 binds to the Hh receptor PTCH to activate the GLI1 zinc finger transcription factor. GLI1 translocation to the nucleus activates the transcription of the anti-apoptosis target genes such as BCL-2. In the canonical Wnt signaling pathway, *E. chaffeensis* TRP120 directly engages FZD (Frizzled) 5 and recruits coreceptor LRP5/6 (lipoprotein receptor-related protein 5 and 6). Activation of Wnt signaling results in the disassembly of the β-catenin destruction complex [consisting of Axin, APC (adenomatous polyposis coli), GSK3β (glycogen synthase kinase 3 beta), and CK1 (casein kinase)], which allows accumulation of β-catenin in the cytoplasm and subsequent nuclear translocation and activation of Wnt target pro-infection genes and Wnt signaling promotes PI3K (phosphatidylinositol 3-kinase)/AKT signaling and downstream suppression of autophagy. In the Notch signaling pathway, *E. chaffeensis* TRP120 interacts with ADAM17 (a disintegrin and metalloproteinase 17) and the Notch1 receptor, resulting in receptor cleavage and nuclear translocation of NICD (Notch intracellular domain), the transcriptionally active form that interacts with Notch RBPjk (recombinant binding protein suppressor of hairless involved in Notch signaling) and MAML (mastermind-like protein 1) transcription factors proteins. This transcription complex then activates transcription of Notch target genes, resulting in inhibition of ERK1/2 (extracellular signal-regulated kinases) and p38 MAPK (p38 mitogen-activated protein kinase) phosphorylation pathway and the downstream transcription factor PU.1 expression is repressed, inhibiting TLR2/4 expression. **(B)** *E. chaffeensis* TRP120 activates conserved signaling pathways *via* TRP120 Hh-, Wnt- and Notch-specific SLiM ligand mimetics embedded within each TR domain. Combined bioinformatic and functional characterization have identified *E. chaffeensis* TRP120 Hh, Wnt and Notch repetitive SLiMs as shown in blue (Hh), purple (Wnt), and green (Notch), respectively.

A diverse array of host cell proteins involved in cytoskeletal organization, vesicle trafficking, cell signaling, transcriptional regulation, post-translational modifications, autophagy, and apoptosis have been shown to interact with *E. chaffeensis* TRP32, TRP47, TRP75, and TRP120 (Byerly et al., 2021). For example, TRP75 interacts with host cell targets involved in homeostasis, cytoskeleton organization, and apoptosis regulation to promote infection (Luo et al., 2018). Further, transient inhibition of the Wnt, Notch, and Hh signaling with small molecule inhibitors or small interfering RNAs (siRNAs) demonstrated that specific activation of these pathways is required for ehrlichial infection (**Figure 2A**) (Lina et al., 2016; Luo et al., 2016; Lina et al., 2017; Rogan et al., 2019; Byerly et al., 2022; Patterson et al., 2022). The Wnt, Notch, and Hh signaling pathways are evolutionarily conserved eukaryotic signaling cascades that not only regulate proliferation, cell fate and development, but also mediate innate immune mechanisms, including autophagy, cytokine expression, and phagocytosis (Sugimura and Li, 2010; Schaale et al., 2011; Maiti et al., 2012; Kim et al., 2013; Petherick et al., 2013; Zhu and Zhang, 2013). Notably, *E. chaffeensis* TRP120 directly interacts with Wnt, Notch, and Hh pathway receptors and components (Luo et al., 2011; Zhu et al., 2017; Wang et al., 2020; Rogan et al., 2021; Byerly et al., 2022; Patterson et al., 2022). Specifically, *E. chaffeensis* TRP120 activates the Wnt signaling pathway to avoid lysosomal fusion with the *E. chaffeensis*-containing vacuole (Lina et al., 2017). By targeting Wnt signaling, TRP120 activates mTOR and blocks nuclear translocation of TFEB, a transcription factor controlling lysosome biogenesis, thereby inhibiting autolysosome generation and autophagic destruction of *E. chaffeensis* (Lina et al., 2017). TRP120 also activates the Notch signaling pathway, resulting in the inhibition of the extracellular signal-regulated kinase 1/2 (ERK1/2) and p38 mitogen-activated protein kinase (MAPK) pathways and subsequent downregulation of toll-like receptors 2 and 4 (TLR2 and TLR4) (Hao et al., 2007; Welcker and Clurman, 2008; Matsumoto et al., 2011; Lina et al., 2016). TLR2 and TLR4 are pattern recognition receptors (PRRs) that identify molecular patterns of invading pathogens (Mukherjee et al., 2016); hence, their downregulation during ehrlichial infection blunts host immune response to infection. Recent studies demonstrate that specific activation of the Hh signaling by *E. chaffeensis* TRP120 leads to nuclear translocation of GLI1 and transcriptional upregulation of anti-apoptotic genes such as BCL-2 (Rogan et al., 2021; Byerly et al., 2022; Patterson et al., 2022).

TRP120 also post-translationally modifies host proteins and itself is post-translationally modified within the host cytosol. For example, TRP120 binds to F-BOX and WD domain repeating-containing 7 (FBW7) in a *trans* conformation, targeting FBW7 for proteasomal degradation by ubiquitination with K48-ubiquitin chains. The degradation of FBW7 results in increased levels of FBW7-regulated oncoproteins (Notch, MYC, JUN, Cyclin E) leading to the upregulation of oncoprotein regulated genes (Wang et al., 2020). In contrast, SUMOylated TRP120 interacts with polycomb group ring finger protein 5 (PCGF5), a member of the polycomb group (PcG) protein family that can remodel chromatin to effect epigenetic silencing of genes. As a component of the polycomb repressive complex 1 (PRC1), which is a histone ubiquitin ligase (Piunti and Shilatifard, 2021), PCGF5 stimulates the PCGFs degradation and the upregulation of PRC1-associated *HOX* genes, which promote ehrlichial infection (Mitra et al., 2018).

Notably, sequence-specific ligand-receptor interactions between TRP120 and Wnt, Notch, and Hh receptors activate these pathways to promote ehrlichial infection (**Figure 2B**) (Rogan et al., 2021; Byerly et al., 2022; Patterson et al., 2022). The short linear motifs (SLiMs) of TRP120 interact with Wnt, Notch, and Hh receptors, revealing a ligand mimicry strategy to exploit host cell signaling for infection (Rogan et al., 2021; Byerly et al., 2022; Patterson et al., 2022). Functional investigations using mutant SLiMs and SLiM-targeted antibodies have confirmed that the TRP120 ligand SLiMs indeed activate Hh, Wnt, and Notch signaling pathways (Rogan et al., 2021; Byerly et al., 2022; Patterson et al., 2022). *E. chaffeensis* TRP120 activation of multiple conserved signaling pathways to promote infection emphasizes an example of the key role of T1SS effectors in pathobiology and provides a useful research model to further investigate T1SS effectors in obligate intracellular bacteria.

## Nucleomodulins

The most extensively studied of the Rickettsiales T1SS substrates are the multiple *E. chaffeensis* effectors that were initially characterized as nucleomodulins. These effectors are capable of binding host DNA through protein–DNA complexes and interacting with host targets to modify chromatin epigenetics. Within the nucleus, TRP32 binds G-rich motifs with GGTGGC-like sequence repeats and targets genes that mediate cell signaling, transcription, cell proliferation/differentiation, and apoptosis (Farris et al., 2018). TRP47 translocates to the nucleus *via* a MYND-binding domain-dependent mechanism (Kibler et al., 2018). Further, investigation has shown that TRP47 predominantly binds enhancers of host genes associated with signal transduction, cytoskeletal organization, and immune response (Kibler et al., 2018). As a nucleomodulin, TRP120 binds to GC-rich regions of DNA in an ordered structure to form a protein–DNA complex, resulting in the regulation of multiple host cell functions, including cell signaling, cytoskeletal organization, transcription, translation and apoptosis (Klema et al., 2018; Byerly et al., 2021). Ank200 also translocates to the nucleus and binds adenine-rich *Alu* elements, which are responsible for regulation of tissue-specific genes, in host promoter and intron regions. The functional categories of identified target genes include transcriptional regulation, apoptosis, ATPase activity, and nuclear structure (Zhu et al., 2009). Interestingly, different mechanisms related to post-translational modifications have been shown to regulate the transcriptional effects and effector-host interactions of *E. chaffeensis* nucleomodulins. For example, tyrosine phosphorylation is required for the nuclear localization of *E. chaffeensis* TRP32 (Farris et al., 2016), while ubiquitination and SUMOylation are critical for TRP120 effector interactions and functions in the host nucleus (Dunphy et al., 2014; Zhu et al., 2017).

Studies of other Rickettsiales members have further solidified the role of T1SS effectors in acting as nucleomodulins to enhance infection. Specifically, *O. tsutsugamushi* Ank1 and Ank6 effector proteins are transported into the host cell nucleus *via* the classical importin-dependent pathway β1 (Evans et al., 2018). In the nucleus, Ank1 and Ank6 induce export *via* exportin 1 of the p65 subunit of NF-κB from host nucleus, thereby reducing the accumulation of p65 in the nucleus. This process ultimately inhibits the transcription of pro-inflammatory genes of the NF-κB pathway (Evans et al., 2018). Similarly, *O. tsutsugamushi* Ank13 nucleomodulin co-opts the eukaryotic RaDAR (RANGDP-ankyrin repeats) nuclear import for nuclear translocation (Adcox et al., 2021). Further, *O. tsutsugamushi* Ank13 downregulates over 2,000 host genes, which are involved in the inflammatory response, transcriptional control, and epigenetics (Adcox et al., 2021). These findings illustrate how *O. tsutsugamushi* employs multiple T1SS effectors to manipulate the expression of host targets in the nucleus to reprogram host transcription to promote infection.

## *Ehrlichia* T1SS immunoreactive proteins

In the previous section, we discussed how *Ehrlichia* T1SS effectors are potent inhibitors of the host response to infection. Interestingly, data demonstrate that *Ehrlichia* T1SS effectors function as major immunoreactive proteins that potentially induce a protective immune response (McBride and Walker, 2010). A small subset of T1SS substrates (TRPs/Anks) have been the primary focus of studies designed to understand antigens involved in protective immunity (Doyle et al., 2006; McBride et al., 2007; Luo et al., 2008; Luo et al., 2009; McBride et al., 2011). Recent investigations in the *E. chaffeensis* and *E. canis* ORFeomes have expanded the list of immunoreactive T1SS effectors which have conformation-dependent antibody epitopes (Nethery et al., 2007; Luo et al., 2010; McBride and Walker, 2011). However, various factors have impeded systematic investigation of immunoreactive proteins in *Ehrlichia*, including the lack of efficient approaches to identify antigen candidates *in silico* and a suitable system to express full length *Ehrlichia* proteins in the native conformation. A high-throughput antigen discovery strategy combining genomics, bioinformatics, cell-free protein expression, and immunoscreening approaches have overcome those obstacles, and led to the identification of undiscovered antigens from the genome of *Ehrlichia* species (Luo et al., 2020; Luo et al., 2021). In particular, the bioinformatic tool ANTIGENpro, a sequence-based predictor of protein antigenicity, was applied to identify antigenicity candidates from *E. chaffeensis* and *E. canis* ORFeomes. These candidates were then subjected to a systematic immunoreactivity screening by using an *in vitro* transcription and translation (IVTT) system which can produce soluble and native recombinant proteins for functional studies (Shimizu et al., 2006; Carlson et al., 2012). Next, the immunoreactive candidates were validated using human monocytic ehrlichiosis (HME) patient sera and canine monocytic ehrlichiosis (CME) naturally infected dog sera, and further investigated for their intrinsic characteristics, including immunoreactivity comparison with “gold standard” TRPs, conformational immunoreactivity, bioinformatic prediction for transmembrane, secretion, and effector. By using this advanced approach, a total of 164 and 69 ehrlichial proteins, many that are small (≤ 250 aa) and predicted to be T1SS substrates, have been identified as immunoreactive proteins in the *E. chaffeensis* and *E. canis* ORFeomes, respectively (Luo et al., 2020; Luo et al., 2021). These newly identified immunoreactive proteins are targets for further molecular characterization that may lead to new diagnostics and subunit vaccines for ehrlichioses.

**Figure 3:**
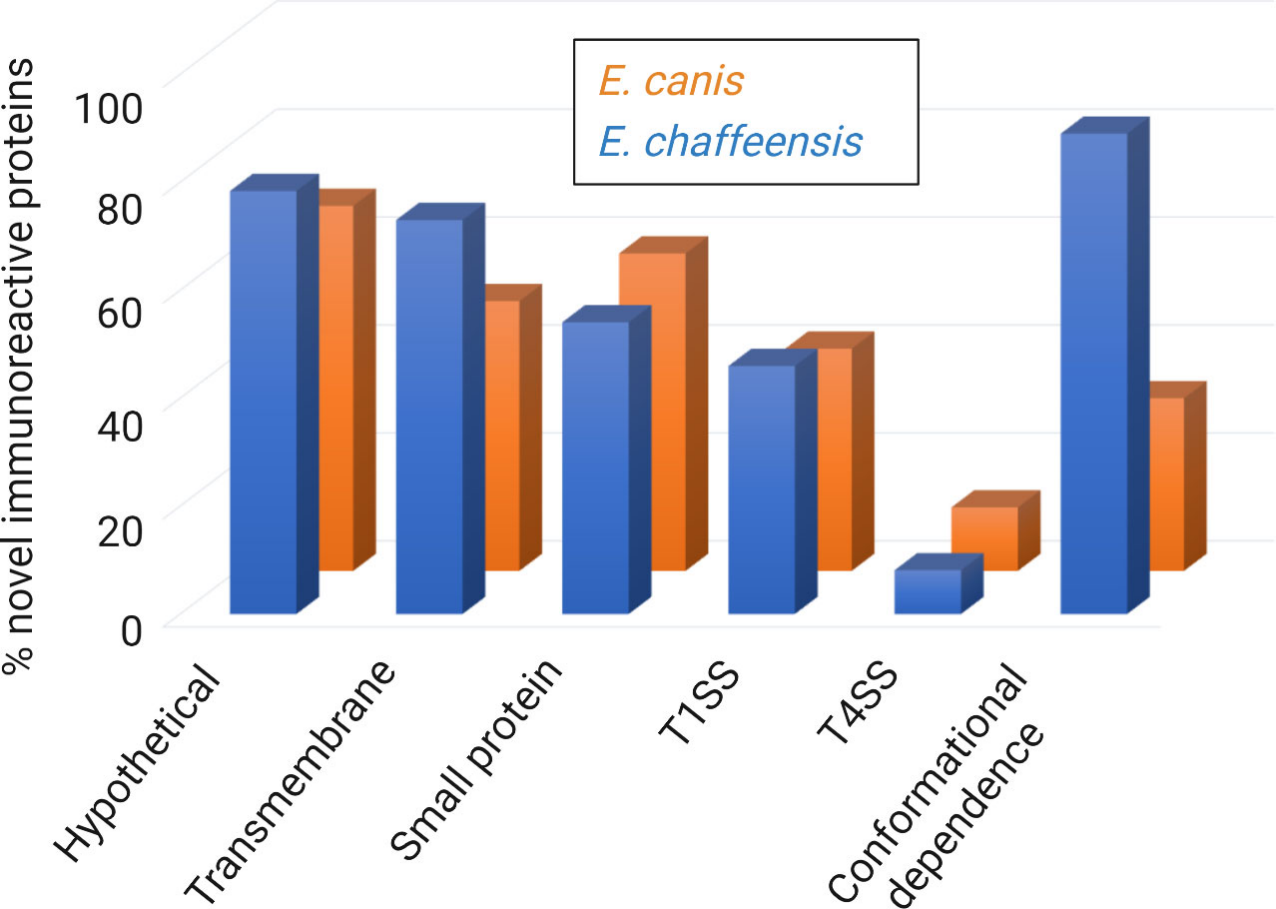
Summary of major predicted features of new immunoreactive proteins from *E. chaffeensis* and *E. canis* ORFeomes. Major immunoreactive proteins from *E. chaffeensis* and *E. canis* ORFeomes (complete set of open reading frames in the genome) are hypothetical, predicted to be T1SS or T4SS effectors, and have conformational antibody epitopes. The secretion system prediction was obtained by comprehensive bioinformatic analysis using multiple effector prediction tools. The conformation-dependent antibody epitopes have been experimentally demonstrated in most proteins.

A key to identifying the major immunoreactive proteins is to determine the epitopes that their antibodies recognize. Epitopes (also known as binding sites) are generally divided in two categories, linear epitopes where a stretch of continuous amino acids are sufficient for binding and conformational epitopes where key amino acid residues are brought together by protein folding (Forsström et al., 2015). A number of prediction systems for linear and conformational epitopes have been developed with some accuracy (Lo et al., 2021). However, these predicted candidates must be experimentally consolidated to demonstrate their proper action. The *Ehrlichia* immunomes (the *Ehrlichia* proteome that reacts with antibody) has been systematically investigated using the aforementioned approach of both *in silico* prediction and experimental verification (Luo et al., 2020; Luo et al., 2021). Notably, a large proportion of new *E. chaffeensis* and *E. canis* immunoreactive proteins has been experimentally demonstrated to harbor conformational antibody epitopes (**Figure 3**). These results indicate that the *Ehrlichia* immunomes have a predominance of epitopes with conformation-dependence. Further, these new findings not only explain previous challenges in identifying *Ehrlichia* immunoreactive proteins, but also establishes an approach to study antigenic proteins and protective immune responses to *Ehrlichia*, whereby conformation-dependence and host-specific (tick vs. mammalian) are considered.

Comprehensive immunomolecular analysis has revealed that many new immunoreactive *Ehrlichia* proteins are small (≤ 250 aa), predicted to contain transmembrane domains, and are T1SS substrates (**Table 3** and **Figure 3**) (Luo et al., 2020; Luo et al., 2021). Further, while both T1SS and T4SS effector proteins play important roles in ehrlichial pathobiology, this suggests that *Ehrlichia* predominantly utilizes T1SS effectors to exploit host cell signaling and transcription to evade innate immune defenses and promote intracellular survival. Taken together, these findings highlight previously unknown roles of *Ehrlichia* T1SS effectors in pathobiology and immunity and establish a model for further investigation of the molecular features of protective proteins, whereby the predicted *Ehrlichia* T1SS effectors would be high priority targets of a protective host immune response.

## Concluding remarks

The T1SS is widely employed by bacteria to secrete exotoxins and effectors, and the T1SS components are conserved among the obligate intracellular bacteria in the order Rickettsiales. However, the molecular function of T1SS components and associated effector substrates has only been experimentally investigated in the genera of *Ehrlichia* and *Orientia*, whereby these T1SS effectors appear to be required for reprogramming the host cell to circumvent the innate defenses. Notably, *Ehrlichia* T1SS TRP effectors, as moonlighting proteins, have been demonstrated to engage multiple host cell processes, including cell signaling, cytoskeletal organization, vesicle trafficking, transcriptional regulation, post-translational modifications, autophagy, and apoptosis. Activation of conserved signaling pathways *via* ligand mimicry has emerged as a key mechanism for host-*Ehrlichia* interactions, with the *E. chaffeensis* TRP120 effector providing a molecularly defined model to investigate SLiM mimicry in pathobiology of obligate intracellular bacterial pathogens. Multiple *Ehrlichia* and *Orientia* T1SS effectors function as bacterial nucleomodulins to modulate host cell survival-associated genes that ultimately promote infection and evade innate immune response. Unexpectedly, many of the newly defined, conformation-dependent immunoreactive proteins are predicted to be T1SS effectors, providing crucial insight regarding proteins and epitopes that may generate a protective immune response. Understanding the molecular role of these interactions over the course of infection will likely lead to the development of new antimicrobial and immunomodulatory therapeutics as well as subunit vaccines for the obligate intracellular bacteria.

## Author contributions

D-CB drafted the manuscript. TL contributed to the editing of the manuscript. JWM organized, directed, and contributed to the writing and editing of the manuscript. All authors contributed to the article and approved the submitted version.
